# The impact of the social adaptability of different groups on the human capital level in an uncertain environment: Panel data analysis based on 35 urban life satisfaction

**DOI:** 10.3389/fpsyg.2022.990941

**Published:** 2022-10-26

**Authors:** Shengwen Yan, Xiaowen Li, Tianan Yan

**Affiliations:** ^1^Institute of Management, Beijing Academy of Social Sciences, Beijing, China; ^2^School of Insurance and Economics, University of International Business and Economics, Beijing, China; ^3^National Engineering Center of Science and Technology Information, Institute of Scientific and Technical Information of China, Beijing, China

**Keywords:** social adaptability, life satisfaction, human capital level, uncertainty environment, QRPD model

## Abstract

To explore the role of the social adaptability of different groups in human capital in an uncertain environment, this paper empirically tests the relationship between social adaptation and human capital by using a panel quantile regression model (QRPD) with the life satisfaction of 35 cities in China as an indicator. By selecting Nelder Mead and adaptive MCMC estimation methods, this paper finds that (1) the higher the level of social adaptation is, the more obvious the promotion effect on the level of human capital; (2) for eastern cities, social adaptation can promote the improvement of the human capital level; for central or western cities, the relationship between social adaptation and human capital is not obvious and depends on the method selected for estimation. To enhance the social adaptability of individuals, the government leadership should actively create conditions, carry out targeted social adaptation training and active adaptation skills exchange for different social groups in order to strengthen social adaptability and enhance human capital. At the same time, policymakers need to consider the differences between the eastern, central and western regions and choose the most suitable human capital improvement action framework for the region to manage environmental uncertainty and make regional human capital sustainable competitive in the era of uncertainty.

## Introduction

Since 2020, the epidemic of COVID-19 infection with pneumonia has spread repeatedly in many places in China. China has taken intensive measures to support epidemic prevention and the resistance war, and the Chinese people have worked together to combat the epidemic. To avoid large-scale population mobility and aggregation, prevention and control measures, such as home isolation and extended holidays, have been taken, forming a governmental, enterprise and social response network for epidemic control. The epidemic has interfered with the normal operation of the labor market. Under the background of great downward pressure on the economy, there may be accelerated relocation of the industrial chain, accelerated spread of operational difficulties of small and medium-sized enterprises, increased employment pressure, and a rapid rise in the unemployment rate (Lee et al., 2021). COVID-19 has had a new impact on the economic development and employment of all regions (Stead et al., 2022). These impacts are mainly reflected in the risk that short-term unemployment and structural unemployment will evolve into permanent unemployment, the increased risk of instability in the employment demand of enterprises, the instantaneous pressure of small and microenterprises where groups with employment difficulties converge, the panic of college graduates when facing their first employment opportunities, and an overload of public employment services (Zhuang et al., 2021).

With the continued recurrence of the epidemic, everyone, every industry, and every node of the urban system are deeply aware of the impact of the era of uncertainty. Some scholars call this era the vuca era and apply it to the business field (Bennett and Lemoine, 2014). Vuca refers to the era of v = volatility, u = uncertainty, c = complexity and a = ambiguity. These changes caused by other unforeseeable changes, due to uncertainty, and complex, fuzzy and interrelated due to various force factors are the current and future oriented state. Facing an uncertain environment, enhancing the social adaptability of different groups, improving the level of workers’ human capital, and securing the bottom line of employment and people’s livelihood are major challenges that need to be addressed (Ramakrishnan, 2021).

With the rapid development of the economy and the increasing enrichment of material culture, health issues have become the focus of attention worldwide. In the report of the 19th CPC National Congress in October 2017, it was proposed that China implement the healthy strategy. Under the guidance of the “Healthy China 2030” strategy, China has entered a new era of assuming the health of all people, and human capital, as the internal output and ultimate goal of health, is necessary to realizing the transformation of China from a populous country to a powerful country in human resources. The World Bank pointed out in the 1993 world development report that good health can lead to improvement in individual labor productivity and can increase the economic growth rate of all countries (Bank, 1993). The World Health Organization (1990) pointed out that an individual can be called a completely healthy individual only if they are sound in four aspects: physical health, mental health, good social adaptability and morality (Chirico, 2016).

In existing research on the relationship between health and human capital, there are two deficiencies. First, there are few studies on the impact of health on human capital from the perspective of social adaptation. Although many scholars at home and abroad have also brought health into the framework of human capital to analyze its impact on economic growth from micro and macro perspectives, most domestic and foreign studies have discussed the impact of human capital from the perspective of individual physical and mental health and morality, ignoring the impact of the perspective of social adaptation (Jin, 2022). The physiological health level includes the impact of nutrition intake (Schultz, 1997; Wang Yin, 2009), height (Schultz, 2002), and body mass index (Sabia and Rees, 2012; Jiaqi, 2016) on wages. The mental health level includes the impact of psychological disorders (Ettner et al., 1997; Lihua, 2017) and depression (Kessler et al., 2006) on labor productivity or wage rate. The moral level includes the impact of such traits as fairness (Manning, 1993) and the determination of capitalist wages by moral factors at the macro level (Zhiyuan, 2008), etc. Social adaptation, as an important indicator of individual mental health, was first proposed by Spencer (1902), which means that individuals gradually accept social moral norms and codes of conduct through self-regulation in the interaction of the social environment and finally achieve a state of psychological and social coordination. At the micro level, social adaptation can bring a sense of security to individuals or families, which is the basis of workers’ learning ability and intellectual and physical strength and has an impact on individual human capital (Parcel and Dufur, 2001). Therefore, as an important part of health and a member of the index system of health human capital, the role of social adaptation on the level of human capital deserves an in-depth study.

Second, the existing research on the relationship between social adaptation and human capital only focuses on the social adaptation and human capital of special groups. The adaptation process of immigrants entering the new living and working environment is also called social integration. Scholars at home and abroad have discussed the relationship between social integration and human capital. Chiswick (1978) found that immigrants’ income was always lower than that of local whites at the initial stage of migration. With the accumulation of labor experience, their income increased faster and was equal to or higher than that of local people 10 to 15 years later. Considering this phenomenon, subsequent scholars explored the gradual narrowing of the income gap of immigrants and the process and mode of income change among different immigrant groups from the vertical and horizontal perspectives (Borjas, 1982, 1985; Borjas and Bronars, 1992) and explored and found that human capital plays an important role in the social adaptation and psychological identity of the floating population in the destination. It is proposed that human capital should be applied to the study of immigrants’ economic status (Yang Juhua, 2016). This study believes that the process of adaptation and integration of immigrant groups, as a part of the whole society, reflects social adaptation to a certain extent but does not provide insight into all human resources.

Therefore, in view of the social adaptation of social members, which is an important indicator of health, this paper discusses its functional characteristics on human capital in an uncertain environment to clarify the role of social adaptation of different groups in the formation and accumulation of human capital, provide policy guidance for relevant departments to improve the social adaptability of social members, and then improve labor supply.

## Research methods

### Variables and measurements

#### Social adaptation measurement

In recent years, scholars have taken life satisfaction as an indicator for measuring social adaptability, discussed the influencing factors of social adaptability (Jiménez Ambriz et al., 2012), and empirically concluded that life satisfaction can predict social adaptability (Li Lina et al., 2014). Vera Toscano proposed that housing satisfaction, as one of the determinants of overall life satisfaction, has a significant correlation with social adaptation (Vera-Toscano and Ateca-Amestoy, 2008). Therefore, in this paper, life satisfaction represents individual social adaptation.

#### Measurement method of human capital level

Scholars have great differences in the methods used to measure the human capital level. At present, the widely used methods include the education years method, value investment estimation method, production function wage method, human capital return method and income relationship method. Bai Peiwen used these methods to calculate and compare and believed that the relative index of the human capital stock level calculated under the production function wage method was more reasonable (Peiwen, 2010). In addition, because this paper Studies 35 major cities in China and the current statistical information at the city level in China is not comprehensive compared with the provincial and national information, the production function wage method is based on the availability of data at the city level in China to measure human capital, which is suitable for the research object of this paper. Therefore, this paper selects the production function wage method as the measurement method of the human capital level.

The production function wage method was proposed by Zhu Pingfang and Xu Dafeng to expand the human capital estimation method based on labor income (LIHK; Zhu and Xu, 2007). Under the assumption of the C–D production function, let w(h) be the wage of workers, and the parameter β The marginal productivity theory combined with production function $Y=K^{1-\beta }{\left(AH\right)}^{\beta }$and factor reward can be deduced and expressed as the share of labor income in total income, that is$\beta =\frac{w\left(h\right)L}{Y}$, let A_1_ be the technical level of the economy formed by unit human capital, which is a constant, w (1) is the wage level of unit human capital, K is the amount of *per capita* material capital, and let w^*^ (1) be the efficiency wage of unit human capital, then $w^{*}\left(1\right)=\frac{w\left(1\right)}{A_{1}^{\beta }}=\beta k^{1-\beta }$. The wage level of unit human capital is$w\left(1\right)=\beta A_{1}^{\beta }k^{1-\beta }$, and the technical level of the economy with human capital h is A_h_, then $A_{h}=hA_{1}$. After derivation, we can obtain the estimation formula of human capital: $h_{e}={\left[w\left(h\right)/w^{*}\left(1\right)\right]}^{1/\left(2\beta \right)}$ (assuming the combination of technology and human capital). Therefore, the estimation of human capital can be measured by labor wages, the share of labor income in total income and *per capita* material capital.

When calculating the *per capita* physical capital of cities, this paper uses the method proposed by Zhu Pingfang and Xu Dafeng to calculate the provincial capital output ratio through the provincial physical capital stock data (Zhang Jun and Jipeng, 2004) and GDP and assumes that the capital output ratio of each city is the same as the provincial data used to calculate the *per capita* physical capital K of each city. The depreciation rate is 0.1 (Xueqing, 2013).

### Data selection description

Based on the questionnaire and satisfaction data in the “China urban basic public service satisfaction evaluation and development report” issued by the Marxism Research Institute of the Chinese Academy of Social Sciences in July 2011, this paper constructs a panel model of 35 major cities across the country to study the impact of social adaptability represented by urban basic service satisfaction on the level of human capital from 2011 to 2020. The total number of responses in this satisfaction questionnaire is more than 20,000 every year, and the construction of the evaluation index system is standardized. Therefore, we believe that it is reasonable to select its scientifically calculated satisfaction score as an index to measure social adaptation. The data sources of human capital variables are the database of the National Bureau of Statistics, the statistical database of the China economic network, and the statistical yearbooks of cities over the years.

This paper selects 35 of the 36 major cities identified by the China Bureau of Statistics (Lhasa will not be considered temporarily due to incomplete data), covering the provincial capitals in eastern, central and western China and cities under separate planning. There are certain differences in the environment, basic medical care, education and housing between cities. However, this does not mean that the greater the investment in medical care and education, the higher the satisfaction, and the higher the satisfaction with life does not mean the higher the level of human capital. Finally, this paper compares the different effects of social adaptation on human capital represented by life satisfaction in eastern, central and western China.

### Descriptive statistics of variables

The descriptive statistics of the variables are shown in Table 1:

**Table 1:** Descriptive statistics of variables.

Variable	Observed value	Mean value	Standard deviation	Minimum value	Maximum value
Human capital	210	99.41248	4.599909	92.04061	118.137
Social adaptation level	210	58.15519	3.588462	48.53	68.35

## Empirical process

### Theoretical basis

In this paper, the panel data quantile regression method (QRPD) proposed by Powell is used to regress the data. Specifically, similar to Chernozhukov and Hansen (2005), QRPD adopts a latent variable framework: $Y_{\mathrm{it}}^{d}=q\left({\mathrm{d,U}}_{\mathrm{it}}^{d_{*}}\right)$. Here, $U_{\mathrm{it}}^{d_{*}}\sim U\left(0,1\right)$ indicates the ability or tendency of the result variable, which may be a function of several random interference terms, constants or time variables, and the relationship with the fixed effect is arbitrary (Powell, 2022). In the selection of the quantile processing effect (QTEs), QRPD can estimate this effect; even if the instrumental variable is related to$U_{\mathrm{it}}^{d_{*}}$, making $U_{\mathrm{it}}^{d_{*}}|Z_{\mathrm{it}}≁U\left(0,1\right)$, the structure quantile function can be expressed as:


(1) $$S_{Y}\left(\tau \mathrm{|d}\right)=q\left(\mathrm{d,}\tau \right),\tau \in \left(0,1\right)\;$$

It is worth noting that, unlike QRPD, the structural quantile function of the additive fixed effects model cannot be expressed by Formula (1); that is, the influence of the processing variable (d) on the result variable ($Y_{\mathrm{it}}$) cannot be directly estimated. Specifically, the comparison between QRPD and other quantile estimation methods is shown in Table 2.

**Table 2:** Comparison of models (Maestas et al., 2016).

Compare content	Mixed IVQR	Additive fixed effect	Panel quantile regression(QRPD)
hypothesis	$U_{\mathrm{it}}^{*}|Z_{\mathrm{it}}\sim U\left(0,1\right)$	$U_{\mathrm{it}}|Z_{\mathrm{it}},\alpha_{i}\sim U\left(0,1\right)$	$U_{\mathrm{it}}^{*}\left|Z_{\mathrm{it}}\sim U_{\mathrm{is}}^{*}\right|Z_{i}$
Target model	$Y_{\mathrm{it}}=D_{\mathrm{it}}^{'}\beta \left(U_{\mathrm{it}}^{*}\right)$	$Y_{\mathrm{it}}=\alpha_{i}+D_{\mathrm{it}}^{'}\beta \left(U_{\mathrm{it}}\right)$	$Y_{\mathrm{it}}=D_{\mathrm{it}}^{'}\beta \left(U_{\mathrm{it}}^{*}\right)$
Distribution of outcome variables	$Y_{\mathrm{it}}$	$Y_{\mathrm{it}}-\alpha_{i}$	$Y_{\mathrm{it}}$
Structural quantile function	d'β(τ)	$\alpha_{i}+\mathrm{d'}\tilde{\beta }\left(\tilde{\tau }\right)\;$	d'β(τ)
τ Quantile interpretation	U∗'sτQuantile	U ‘sτQuantile	U∗'sτQuantile

Referring to the model framework of Chernozhukov and Hansen, the QRPD model needs to meet the following conditions (Chernozhukov and Hansen, 2006):

1. Potential results and monotonicity assumption: $Y_{\mathrm{it}}^{d}=q\left({\mathrm{d,U}}_{\mathrm{it}}^{d_{*}}\right),U_{\mathrm{it}}^{d_{*}}\sim U\left(0,1\right)$，q(d,τ) about *τ* Strictly increasing (Chernozhukov and Hansen, 2005);
2. Independence assumption: for all s, t and each D, there is:

$$E\left[1\left(U_{\mathrm{it}}^{d_{*}}\leq \tau \right)-1\left(U_{\mathrm{is}}^{d_{*}}\leq \tau \right)|Z_{i}\right]=0;$$

3. Optional assumption: for some unknown functions $\delta_{t}\left(\cdot \right)$ and random vector$V_{i}$. All$D_{\mathrm{it}}=\delta_{t}\left(Z_{i}{\mathrm{,V}}_{i.}\right)$
4. Sorting similarity: for each $d,d^{\prime }$，$U_{\mathrm{it}}^{d_{*}}\left|Z_{i}{\mathrm{,V}}_{i}\sim U_{\mathrm{it}}^{d_{*}^{\prime }}\right|Z_{i},V_{i}$;
5. Observability: observable random vectors include $Y_{\mathrm{it}}=Y_{\mathrm{it}}^{D},D_{\mathrm{it}},Z_{\mathrm{it}}$;

In addition, the recognition assumptions of the QRPD model are as follows:

6. Assumption of the first stage: The rank of $E\left(Z_{i}\Pi \left(Z_{i}\right)\right)$ is M;

Among them, $\Pi \left(Z_{i}\right)=\left[\begin{array}{ccc} P\left(D_{i1}=d^{\left(1\right)}{\mathrm{|Z}}_{i}\right) & \cdots & P\left(D\_i1=d^{\left(M\right)}{\mathrm{|Z}}_{i}\right)\\ \vdots & \ddots & \vdots \\ P\left(D_{\mathrm{iT}}=d^{\left(1\right)}{\mathrm{|Z}}_{i}\right) & \cdots & P\left(D_{\mathrm{iT}}=d^{\left(M\right)}{\mathrm{|Z}}_{i}\right) \end{array}\right]$

7. Given $Z_{i}$，$Y_{\mathrm{it}}$ It is a continuous distribution.

If (1) assumption, I–VII is true;


(2) $$E\{\frac{1}{T}\sum_{t=1}^{T}\left(Z_{\mathrm{it}}-\bar{Z_{i}}\right)[1\left(Y_{\mathrm{it}}\leq \tilde{q}\left(D_{\mathrm{it}},\tau \right)\right]\}=0;$$

(3) $$E[1(Y_{\mathrm{it}}\leq \tilde{q}\left(D_{\mathrm{it}},\tau \right)=\tau \mathrm{then}\tilde{q}\left(D_{\mathrm{it}},\tau \right)=q\left(D_{\mathrm{it}},\tau \right)\;$$

Based on the QRPD method, the human capital and social adaptation data of 35 cities from 2011 to 2016 were used for regression, and 9 quantiles were selected to estimate the model using the Nelder Mead estimation method (the default method) and MCMC estimation method, respectively.

### Introduction of MCMC method

The Markov chain Monte Carlo (MCMC) method is usually used to extract samples from probability distribution functions that are difficult to invert or have complex standardized constants. This method was first used in Bayesian analysis and estimation and later used in many estimation problems. Chernozhukov and Hong (2003) believe that the MCMC method can be applied to many traditional statistical inference problems (Chernozhukov and Hong, 2003). Specifically, it can be applied to any model with a “pseudo quadratic” objective function, including many models using maximum likelihood or GMM estimation. The MCMC estimation method of QRPD uses the “adaptive MCMC” algorithm.

The core of MCMC sampling is the metropolis Hastings (MH) algorithm, which is constructed around a sampling target distribution function π(X) and a proposed distribution function q(Y,X). If we mainly focus on MCMC estimation, π(X)can be regarded as a conditional likelihood function, and X is the parameter vector of 1×n. The basic MH algorithm can be expressed as follows:

1. Initialization: $X=X_{0}$, time T;
2. Set t = 0, and repeat 3–6 when t≤T;
3. Extract candidate $Y_{t};$ from $q\left(Y_{t}{\mathrm{,X}}_{t}\right)$
4. calculation $\alpha \left(Y_{t}{\mathrm{,X}}_{t}\right)=\min\left\{\frac{\pi \left(Y_{t}{\mathrm{,X}}_{t}\right)}{\pi \left(X_{t}\right)}\frac{q\left(Y_{t}{\mathrm{,X}}_{t}\right)}{q\left(X_{t}{\mathrm{,Y}}_{t}\right)},1\right\}$
5. with $\alpha \left(Y_{t}{\mathrm{,X}}_{t}\right)$ probability setting $X_{t+1}=Y_{t}$. With $1-\alpha \left(Y_{t}{\mathrm{,X}}_{t}\right)$ probability setting $\;X_{t+1}=X_{t}$;
6. Gradually increase t, and finally get $\;{\left(X_{t}\right)}_{t=1}^{T}$ series.

The MCMC sampling method is flexible, but it is difficult to implement, mainly because it is difficult to select the appropriate recommended distribution function. If the proposed distribution function is selected poorly, the coverage of the target distribution function is relatively poor, so it is more appropriate to select “adaptive MCMC,” which can adjust the proposed distribution function. An MH adaptive algorithm based on the symmetry proposed distribution function is as follows:

1. Initialization: $X=X_{0}$, time T;
2. Set t = 0, and repeat 3–7 when t≤T;
3. Extract candidate $Y_{t};$ from $q\left(Y_{t}{\mathrm{,X}}_{t},\theta_{t}\right)$
4. calculation $\alpha \left(Y_{t}{\mathrm{,X}}_{t}\right)=\min\left\{\frac{\pi \left(Y_{t}{\mathrm{,X}}_{t}\right)}{\pi \left(X_{t}\right)},1\right\}$
5. with $\alpha \left(Y_{t}{\mathrm{,X}}_{t}\right)$ probability setting $X_{t+1}=Y_{t}$. With $1-\alpha \left(Y_{t}{\mathrm{,X}}_{t}\right)$ probability setting $X_{t+1}=X_{t}$
6. Updating $\theta_{t+1}=f\left(\theta_{t}{\mathrm{,X}}_{0}{\mathrm{,X}}_{1}{\mathrm{,X}}_{2},\ldots {\mathrm{,X}}_{t}\right)$
7. Gradually increase *t*, and finally get ${\left(X_{t}\right)}_{t=1}^{T}$ series.

### Estimated results

The full sample regression results are shown in Table 3:

**Table 3:** Full sample regression results (*n* = 210).

Quantile	0.1	0.2	0.3	0.4	0.5	0.6	0.7	0.8	0.9
coefficient	0.0146^***^	0.0300^***^	0.0787^***^	0.0979^***^	0.1091^***^	0.1683^***^	0.2069^***^	0.3581^***^	0.5535^***^
T value	2.67	6.36	49.71	20.88	17.38	23.76	17.60	223.64	231.43

^***^, ^**^, ^*^Represented at the 1, 5, and 10% significance levels, respectively, are significant.

From the estimation coefficient, the coefficient is significantly positive at each quantile level; that is, the level of social adaptation can significantly promote the level of human capital. Moreover, a higher level of social adaptation plays a more obvious role in promoting the level of human capital. For example, at the 0.9 quantile level, the regression coefficient is 0.5535, while at the 0.1 quantile level, the coefficient is 0.0146, a difference of nearly 55 times (Table 4).

**Table 4:** Full sample regression results MCMC (*n* = 210).

Quantile	0.1	0.2	0.3	0.4	0.5	0.6	0.7	0.8	0.9
Coefficient	0.0023	0.0319^***^	0.0770^***^	0.0997^***^	0.1212^***^	0.1668^***^	0.2285^***^	0.3607^***^	0.5385^***^
*t*-Value	0.22	5.95	14.52	9.06	8.75	11.10	13.03	21.82	13.96

^***^, ^**^, ^*^Represented at the 1, 5 and 10% significance levels, respectively, are significant.

From the estimation results of MCMC, except for the 0.1 quantile point, the estimation coefficients on the remaining quantiles are similar to the estimation results of the default estimation method (Nelder Mead). Thus, for the whole sample, we can get a similar conclusion that says, “the level of social adaptation can significantly promote the level of human capital. Moreover, the higher the level of social adaptation, the more obvious the promotion effect of human capital.”

### Subsample regression

Due to the huge differences in the level of development among regions in China, the impact of social adaptation on the level of human capital may be different among different regions. Based on this consideration, 35 cities are divided according to the East, middle and West and then grouped for regression. The results are in Table 5:

**Table 5:** Regression results of eastern cities (*n* = 108).

Quantile	0.1	0.2	0.3	0.4	0.5	0.6	0.7	0.8	0.9
Coefficient	0.0438	0.0891^***^	0.1123^***^	0.1356^***^	0.1202^***^	0.1137^***^	0.2651^***^	0.2235^***^	0.1524
*t*-Value	0.53	3.21	28.74	9.22	10.86	3.51	20.94	11.14	0.98

^***^, ^**^, ^*^Represented at the 1, 5 and 10% significance levels, respectively, are significant.

The cities included in the eastern, central and western regions are as follows: (1) Eastern region: Beijing, Tianjin, Shijiazhuang, Shanghai, Nanjing, Hangzhou, Ningbo, Fuzhou, Xiamen, Jinan, Qingdao, Guangzhou City, Shenzhen City, Haikou City, Dalian City, Shenyang City, Changchun City, Harbin City; (2) Central Region: Taiyuan City, Hefei City, Nanchang City, Zhengzhou City, Wuhan City, Changsha City; and (3) Western Region: Hohhot, Nanning, Chongqing, Chengdu, Guiyang, Kunming City, Xi’an City, Lanzhou City, Xining City, Yinchuan City, Urumqi City.

From the regression results of cities in the eastern region, (1) excluding the extreme (extremely low or extremely high) level of social adaptation, the level of social adaptation can significantly promote the growth of the human capital level, and at the extreme level, social adaptation has no significant impact on human capital. (2) There is an optimal level of social adaptation. When it is in the 0.7 quantile of social adaptation, social adaptation has the greatest effect on human capital. (3) Different levels of social adaptation have different effects on human capital Table 6.

**Table 6:** Regression results of eastern cities MCMC (*n* = 108).

Quantile	0.1	0.2	0.3	0.4	0.5	0.6	0.7	0.8	0.9
Coefficient	0.0538^***^	0.0937^***^	0.1206^***^	0.1326^***^	0.1482^***^	0.1511^***^	0.2495^***^	0.2619^***^	−0.6353
*t*-Value	18.88	5.13	5.56	8.44	5.54	2.93	4.38	5.60	−1.25

^***^, ^**^, ^*^Represented at the 1, 5 and 10% significance levels, respectively, are significant.

From the MCMC regression results of cities in the eastern region, (1) except for an extremely high level, the level of social adaptation can significantly promote the growth of human capital, and an extremely high level of social adaptation has no significant impact on human capital. (2) Different levels of social adaptation have different promotion effects on human capital. In general, except for the higher level of social adaptation, social adaptation can significantly promote the growth of human capital, which is similar to the results of the full sample regression (Table 7).

**Table 7:** Regression results of central cities (*n* = 36).

Quantile	0.1	0.2	0.3	0.4	0.5	0.6	0.7	0.8	0.9
Coefficient	−0.0061^***^	−0.0176	−0.0181^*^	−0.0118^***^	−0.0129^***^	0.0238	−0.2164	0.0547^***^	−0.2666
*t*-Value	−3.55	−1.59	−1.76	−10.42	−9.96	1.2	−0.32	3.15	NA

^***^, ^**^, ^*^Represented at the 1, 5 and 10% significance levels, respectively, are significant.

From the regression results of cities in Central China, (1) at different quantile levels, the impact of social adaptation on human capital is different. Generally, a lower level of social adaptation can inhibit human capital (0.1, 0.3, 0.4, and 0.5), while a higher level of social adaptation can promote the growth of human capital. (2) The effect of extremely high social adaptation on human capital is not obvious (Table 8).

**Table 8:** Regression results of central cities MCMC (*n* = 36).

Quantile	0.1	0.2	0.3	0.4	0.5	0.6	0.7	0.8	0.9
Coefficient	−0.0034	0.0080	0.0161	0.0143	0.0195	0.0414^*^	0.0293	0.0365	NA
*t*-value	−0.29	0.27	0.49	0.43	0.61	1.92	1.13	1.62	NA

^***^, ^**^, ^*^Represented at the 1, 5 and 10% significance levels, respectively, are significant.

From the MCMC regression results of central cities, different from the Nelder Mead estimation, except for the 0.6 quantile, the degree of social adaptation has no significant impact on human capital; that is, for central cities, the impact of social adaptation on human capital is uncertain, which is related to the estimation method used (Table 9).

**Table 9:** Regression results of western cities (n = 66).

Quantile	0.1	0.2	0.3	0.4	0.5	0.6	0.7	0.8	0.9
Coefficient	−0.0310	−0.0226^***^	−0.0078	−0.0127^***^	0.0552^**^	0.0700^***^	0.0720^***^	0.0874^***^	−0.0306
*t*-Value	−1.64	−7.35	−0.41	−3.17	2.24	16.19	5.63	5.97	−1.23

^***^, ^**^, ^*^Represented at the 1, 5 and 10% significance levels, respectively, are significant.

From the regression results of cities in the western region, (1) the extreme level of social adaptation has no significant impact on human capital, and (2) lower social adaptation will inhibit human capital (0.1 ~ 0.4), while a higher social adaptation level will significantly promote the level of human capital (Table 10).

**Table 10:** Regression results of western cities MCMC (*n* = 66).

Quantile	0.1	0.2	0.3	0.4	0.5	0.6	0.7	0.8	0.9
Coefficient	−0.0097	−0.0341^*^	−0.0305	0.0063	0.0443^*^	0.0577^***^	0.0542	0.0789	0.0808
*t*-Value	−0.82	−1.65	−0.96	0.19	1.80	4.17	1.51	0.94	0.75

^***^, ^**^, ^*^Represented at the 1, 5 and 10% significance levels, respectively, are significant.

Similarly, the results of MCMC regression show that there is no significant causal relationship between the level of social adaptation and the level of human capital in western cities, except that a moderate degree of social adaptation can promote the level of human capital.

## Discussion

With the normalization of COVID-19 and the arrival of the vuca era, workers need to continue to cope with environmental uncertainty. The conclusion of this study makes a certain theoretical contribution to human capital theory in uncertain environments. The details are as follows: using social adaptation data and human capital data of different groups, this paper empirically tests the relationship between social adaptation and human capital in an uncertain environment through a panel quantile regression model (QRPD). By choosing different estimation methods (Nelder Mead and adaptive MCMC estimation methods), the results show that (1) in general, social adaptation can promote the improvement of the human capital level, and the higher the level of social adaptation is, the more obvious the promotion of the human capital level; (2) for eastern cities, except for the extreme level of social adaptation, social adaptation can promote the improvement of the human capital level; and (3) for central or western cities, the relationship between social adaptation and human capital is not obvious, which depends on the method selected for estimation. If we choose the Nelder Mead method, for central or western cities, lower social adaptation will inhibit human capital, while higher social adaptation will promote the level of human capital. If the adaptive MCMC estimation method is selected, social adaptation will have a significant impact on human capital at individual quantiles and no significant impact on other quantiles. However, in view of the subjectivity of the adaptive MCMC method in the selection of the proposed distribution function, the Nelder Mead estimation will be more reliable. Of course, this method is still developing and needs further research and exploration.

The conclusions of this study also have some implications for the management practice of improving social adaptability. In the workplace, employees may face a rapidly changing environment. This study believes that organizers need to build a systematic strategic action framework to better cope with environmental uncertainty and promote the steady improvement of human capital. The research conclusion has strong policy significance. On the one hand, to enhance the social adaptability of individuals, the government should actively create conditions, carry out targeted social adaptation training and active adaptation skills exchange for different social groups, strengthen the social adaptability of different social groups and individuals at different ages, improve their ability to obtain resources in social activities, promote the accumulation of learning ability and intellectual and physical strength, and enhance their human capital. On the other hand, policies need to take into account the differences between the eastern, central and western regions. In the central and western regions, the relationship between social adaptation and human capital is not consistent with that in the eastern region. Especially in the central and western regions, the government needs to improve communication with local public organizations, strengthen the dissemination of individual social adaptation activities in the eastern and central and western regions, and implement corresponding policies according to local conditions to improve regional human capital.

Furthermore, the conclusions of this study also have some implications for social groups in different development regions to cope with environmental uncertainty. In an uncertain environment, vuca represents four challenges and four solutions: (1) the double high quadrant of volatility, which changes frequently and requires agility. Future social adaptability includes cognitive ability, critical thinking and emotion, social intelligence, etc. These abilities can effectively help labor groups learn quickly and adjust their business direction in time to adapt to new scenarios. (2) The trend of uncertainty is unclear, which requires vision. Leaders with a clear vision have the ability to set good goals for the organization for the next three to five years. It is the existence of vision that enables organizations to ride the storm in an environment full of volatile factors such as economic downturn and endless competitors. Under the guidance of vision, leaders can make decisions to actively respond to changes and lead their organization in the right direction. (3) Complexity is influenced by many factors, which requires understanding. Leaders need to consciously slow down, stop, observe and listen to others carefully in a timely manner, outside the scope of their professional knowledge, to ensure that they can understand the existing and future uncertain factors and make correct decisions under the guidance of vision. In this process, leaders need to communicate with employees at all levels to further develop teamwork at all levels of the company. (4) Ambiguity is a double low quadrant that lacks transparency and requires transparency. Complexity can be resolved through clarification, making chaos meaningful. In the vuca world, chaos becomes swift and elusive. Leaders who can quickly and clearly understand the details related to chaos can make better and wiser business decisions. Moreover, they are open to new behaviors or views and are willing to adapt or abandon existing values when necessary to adapt to the new world. By understanding the social adaptability proposed in this study, as well as the ability differences and action mechanisms of different groups in the east, central and western regions, workers can choose the most suitable human capital improvement action framework for their region to address environmental uncertainty and make regional human capital sustainable competitive in the era of uncertainty.

## Data availability statement

The original contributions presented in the study are included in the article/supplementary material, further inquiries can be directed to the corresponding author.

## Author contributions

SY contributed the central idea, analyzed most of the data, and wrote the initial draft of the paper. XL and TY contributed to refining the ideas, carrying out additional analyses and finalizing. All authors contributed to the article and approved the submitted version.

## Funding

This research was supported by the key project of the Beijing Academy of Social Sciences, “Research on the guiding mechanism of population flow in Beijing under the relief of non-capital functions” (no. 182006); supported by the general project “Research on the construction of Beijing multi-level elderly care service system” of Beijing theoretical system research center of socialism with Chinese characteristics (no. 16ZT004).

## Conflict of interest

The authors declare that the research was conducted in the absence of any commercial or financial relationships that could be construed as a potential conflict of interest.

## Publisher’s note

All claims expressed in this article are solely those of the authors and do not necessarily represent those of their affiliated organizations, or those of the publisher, the editors and the reviewers. Any product that may be evaluated in this article, or claim that may be made by its manufacturer, is not guaranteed or endorsed by the publisher.
